# Challenges of Wearable Biosensors and Ways to Overcome Them

**DOI:** 10.3390/bios16030159

**Published:** 2026-03-13

**Authors:** Sergei Tarasov, Yulia Plekhanova, Anatoly Reshetilov, Sergey Melenkov, Ivan Saltanov

**Affiliations:** 1 G.K. Skryabin Institute of Biochemistry and Physiology of Microorganisms, Pushchino Center for Biological Research of the Russian Academy of Sciences, 142290 Pushchino, Moscow Region, Russia; yu_plekhanova@pbcras.ru (Y.P.); anatol@pbcras.ru (A.R.); 2 MLC GT L.L.C-FZ, Meydan Grandstand, 6th floor, Meydan Road, Nad Al Sheba, Dubai, United Arab Emirates; melenkov.s@mlc.health (S.M.); company@mlc.health (I.S.)

**Keywords:** wearable medical devices, implantable biosensors, continuous glucose monitoring, biosensors, diabetes, glucose biosensors, wearable biosensors, flexible electronics, nanotechnology

## Abstract

In the 21st century, there have been radical changes in healthcare related to the transition from a universal approach to personalized medicine based on the unique characteristics of each patient. In large part, this has become possible due to the development and distribution of wearable medical devices that are capable of providing continuous monitoring of a variety of physiological parameters outside medical institutions. The most important of these devices are modern biosensors that allow real-time tracking of various biomarkers in the body, thereby opening up new opportunities for disease prevention, early diagnosis, and personalized treatment strategies. The most obvious example of the transformation is the implementation of wearable devices for continuous glucose monitoring (CGM), which has significantly facilitated the daily lives of millions of people with diabetes. Nevertheless, despite the examples of successful implementation of these devices, their large-scale distribution is associated with many challenges, such as the need for standardization, data transmission security, and the risks of immune responses to implantable devices or infections. This review examines all the current problems of wearable biosensors and possible ways to overcome them. Special emphasis will be placed on devices for continuous glucose monitoring as the most commercially successful representatives of this device class.

## 1. Introduction

Modern healthcare is undergoing a transition from episodic monitoring of patient conditions directly in clinics to a model of continuous remote monitoring [1]. This transformation is due not only to the insufficient effectiveness of the traditional monitoring model in clinics but also to the growing number of diseases caused by the modern lifestyle and the general aging of the world’s population. The in-door patient monitoring model cannot provide a complete dynamic picture of patients’ vital signs, and, moreover, it is increasingly labor and resource intensive. Therefore, monitoring based on wearable medical devices is increasingly being introduced in modern medicine [2,3]. The key advantage of modern remote monitoring systems is to ensure continuous communication between the patient and the healthcare system, giving medical professionals the opportunity to monitor the real-time dynamics of vital signs in everyday conditions, which was impossible during traditional visits to the clinic. As a result, the effectiveness of the treatment process increases significantly, since decisions can be made based on complete and up-to-date data. This opportunity is becoming especially critical to resolve the problem of inequality in access to medicine for residents of remote and rural areas. The importance of remote monitoring technologies goes beyond simple data collection: they fundamentally change the patient’s interaction with the healthcare system, making them an active participant in the process of managing their own health while reducing the overall burden on healthcare institutions [4,5].

**Figure 1:**
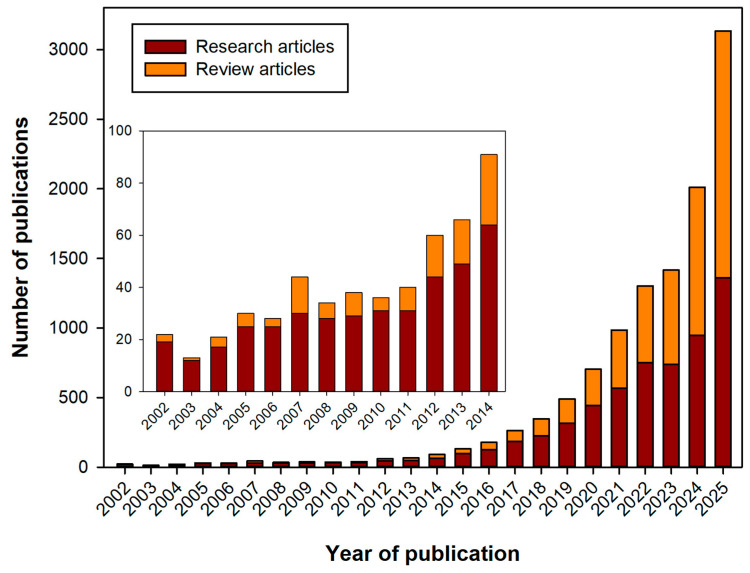
The dynamics of articles including the keywords “wearable biosensors” according to the ScienceDirect database as of November 2025.

The field of remote health monitoring is one of the most dynamically developing at the medicine and technology intersection, so every year, new innovative solutions and devices appear in it. Modern wearable devices equipped with advanced sensors are able to monitor a wide range of health indicators, from heart rate and blood glucose levels to sleep patterns and physical activity [6,7]. They help in the early diagnosis of diseases, facilitate the treatment of chronic diseases, contribute to the development of individual rehabilitation programs, and are a fundamental component of the connected health ecosystems [8]. In recent years, wearable medical devices have increasingly used cutting-edge advances in digital technology, artificial intelligence-based technologies, and the Internet of Things. This allows them to expand their capabilities and further personalize their use, which, in turn, ensures the widespread adoption of these new solutions in the healthcare sector. As a result, the combined volume of the markets for wearable and implantable medical devices is projected to reach 168 billion US dollars in 2030 [9]. The increasing interest in this field every year is reflected in the scientific literature, where the number of studies including the keyword “wearable biosensor” in the last 15 years has shown exponential growth (Figure 1). It is especially worth noting the gradual increase in the proportion of reviews in the last few years, which may indicate a request for systematization and deep understanding of the experimental data obtained for the further development of the industry.

**Figure 2:**
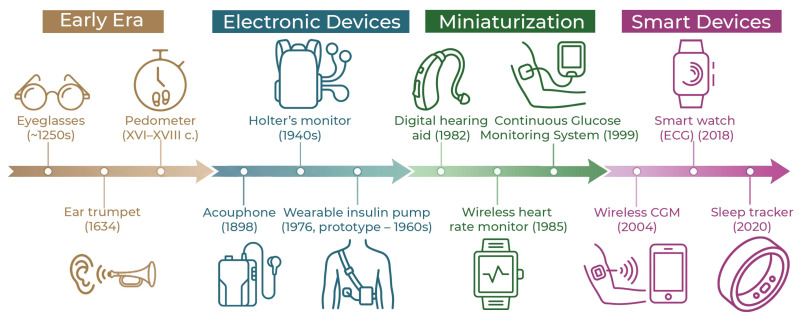
Some milestones in the history of wearable medical device development.

The history of wearable medical devices dates back many decades [10]; some researchers believe that the countdown should be traced back to about the 13th century, when the first wearable devices appeared in Italy to improve eyesight: glasses [11]. Nevertheless, in the modern view, when we talk about WMDs, we mean a wearable medical electronic device (Figure 2 shows the chronology of the development of wearable medical devices). Here, Edward Thorp and Claude Shannon should be considered the pioneers, who, in the 1960s, developed the design of the first wearable computer [12]. Later in the 20th century, the first wearable digital watches, wireless headphones, and even virtual reality eyewear concepts were released [13]. All of these devices were wearable and electronic, but they were not medical. Also, by the end of the 20th century, many electronic medical devices had been developed, of course, the first of which should be considered the Akouphone Hearing Aid, created by Miller Reese Hutchison in 1898 [14]. In the 1940s, the first version of the “Holter monitor” appeared, which allowed continuous outpatient electrocardiography. Nevertheless, due to the size of their electronic parts, these devices could hardly be called truly “wearable”; for example, the first version of the cardiograph, developed by Norman Holter, weighed about 38 kg [15]. Therefore, truly wearable medical devices began to develop closer to the beginning of the 21st century, when it became possible to combine precise medical sensors with compact digital equipment for their processing. It was the miniaturization of wearable devices that allowed them to be considered as devices for clinical monitoring outside the hospital. At first, they were used exclusively for simple procedures such as heart rate counting. The first wireless heart rate monitor, the Polar Sport Tester PE2000, was released in 1985 and immediately gained huge popularity among athletes [16]. In the late 1980s, the first digital hearing aids appeared, developed by Nicolet Corporation and Resound Corporation [17].

In the 1990s, the development of wearable devices began, combining the functions of recording and processing clinical data with telemedicine in the form of real-time wireless telemetry. The pioneers in this direction were NASA, with a program for continuous monitoring of the physiological reactions of astronauts in space, and the US Army, which developed wearable computers for use on the battlefield as part of the Land Warrior program [18]. These devices measured the subject’s blood pressure, pulse, respiration, and blood oxygen levels, but the processing of these data was carried out by external observers and specialists rather than directly by the wearable device. Also in the 1990s, thanks to research in the fields of enzyme biosensors, nanotechnology, and wireless data transmission (or rather the advent of Bluetooth technology, which later became the main one in WMD), it became possible to create devices that would later become the most commercially successful part of the market: continuous glucose monitoring devices [19]. The first FDA-approved device for continuous measurement of glucose in interstitial fluid in 1999 was the MiniMed Continuous Glucose Monitoring System, Dexcom devices entered this market in 2006 and Abbott in 2008 [20]. These early devices were still quite bulky, required frequent calibration, and did not display real-time data, and the sensor’s operating time was no more than 72 h (108 h for the Abbott Freestyle Navigator). But even in this form, they have become a revolutionary step in the treatment of diabetes. In parallel, in the 2000s, the first wearable fitness trackers began to appear, first in the form of chips embedded in shoes (Nike + iPod in 2006) [21] and then in the form of clip-on devices (Fitbit Tracker in 2009) [22] and fitness bracelets. However, at that time, they could strictly be classified as wellness, not medical, devices. In the 2010s, this difference began to blur, because medical wearable devices became more and more autonomous and user friendly, and wellness devices began to include more and more advanced features, thanks to which they were increasingly used not only in everyday life but also in sports medicine. Finally, at the end of the decade, two significant events occurred that forever changed the picture of the market for wearable devices. In 2017, Abbott introduced the Freestyle Libre, the first CGM device that did not require calibration using the classic finger blood sampling method [23]. This marked the transition to truly portable medicine, when a single device is able to take over the functions of a full-fledged diagnostic tool that requires a minimum number of additional actions from the patient. And in 2018, the FDA issued clearance for the use of an electrocardiogram app and an irregular heart rate notification feature on the Apple Watch Series 4 [24]. For the first time, a mass-market consumer device was officially recognized as a medical device. This meant that a single device, such as a smartwatch, could now function both as a wellness product and as a regulated medical device. Despite the fact that from a legal point of view such devices should still be quite strictly delimited by manufacturers according to the areas of their claimed application, they are currently as close as possible from a technological point of view and from the point of view of the software used. We can say that we are now entering the era of hybrid wearable devices, which, within a single platform, organically integrate functions to maintain a healthy lifestyle with the diagnostic and monitoring capabilities of medical devices. In addition, the field of monitoring and therapeutic devices is developing, which not only assess the level of the metabolite but can also independently make decisions about the timely administration of necessary drugs into the patient’s body.

**Figure 3:**
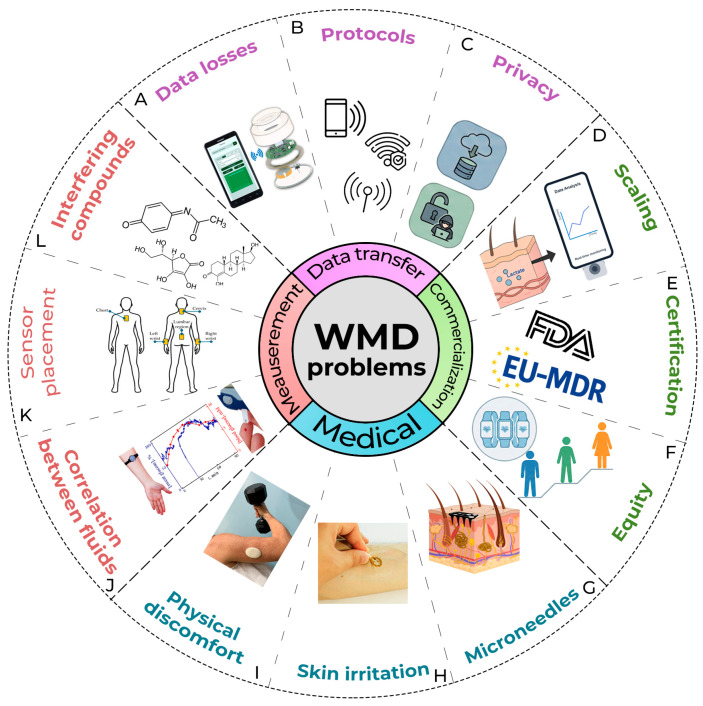
The main categories of problems of wearable medical devices. Schematic representation of sensor components, photos of sensors, and types of signals: (**A**) Reproduced with permission: copyright 2023, American Chemical Society [25]. (**D**) Reproduced with permission: copyright 2025, MDPI [26]. (**G**) Reproduced with permission: copyright 2022, MDPI [27]. (**H**) Reproduced with permission: copyright 2016, AAAS [28]. (**J**) Reproduced with permission: copyright 2019 American Chemical Society [29]. (**K**) Reproduced with permission: copyright 2025, MDPI [30].

Despite all these significant advances in wearable technology, there are a number of critical problems that prevent wearable devices from moving to the next quality level. Most of these problems relate specifically to the “medical” part of WMDs, since the requirements for accuracy, reliability, and safety here are disproportionately higher than in other areas. The existing limitations are most pronounced in the field of medical biosensors, which is especially noticeable in the example of commercially available and developed CGM devices. Even in such a commercially successful field, issues of accuracy, convenience, and autonomy still need to be addressed. This review will classify and examine the existing problems and challenges of wearable and invasive sensors, ways to overcome them, as well as the prospects for this area in the near future (the diagram is shown in Figure 3).

## 2. Medical Problems

When considering wearable biosensor devices for long-term monitoring, it should be understood that they are in contact with a human for quite a long time, so manufacturers need to ensure maximum comfort for those wearing such devices. They can be non-invasive or implantable. Wear comfort is ensured both by the use of materials that do not cause irritation and allergic reactions on the skin and by minimizing injuries when using such devices, both physiological and psychological. Since most of the review is devoted to biosensors that monitor the level of metabolites in the human body, mainly glucose levels, we will focus on their specific problems. We will start with the most “acute” ones, which can lead not only to discomfort when wearing but also to serious consequences for human health.

Any device consists of a number of parts that are interconnected and whose interaction affects the final result. Only large companies with specialists in different fields of knowledge can develop and combine all these parts in one device. Let us look at the individual parts of such devices and the problems associated with their functioning.

**Figure 4:**
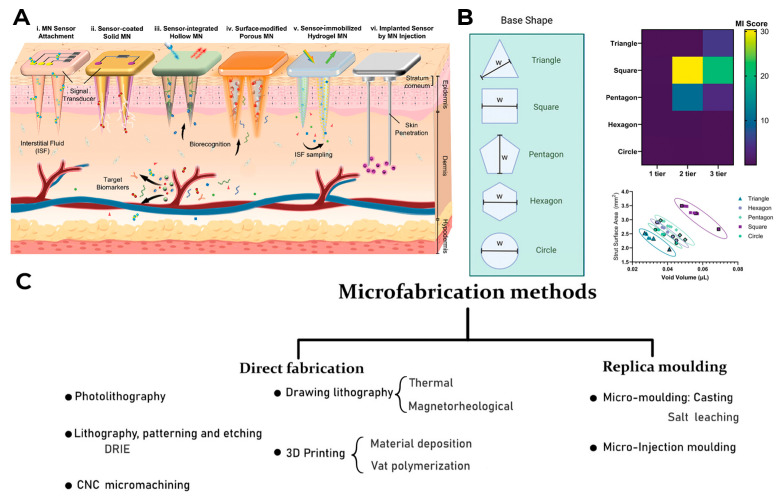
Types of microneedles used in wearable medical devices: (**A**) reproduced with permission: copyright 2024, Wiley [44]; their geometry: (**B**) reproduced with permission: copyright 2024, Wiley [45]; the methods of their creation: (**C**) reproduced with permission: copyright 2025, Wiley [46].

Currently, most devices for monitoring the level of metabolites in the human body are associated with blood and interstitial fluid analysis, where the level of metabolites is approximately the same, and in order to study this level, it is necessary to access this fluid efficiently and reliably or extract it from the body. Needles play an important role in devices for collecting the analyzed liquid. They are the part of monitoring devices that directly penetrates the body and can cause painful sensations and discomfort when worn. While most CGM devices from leading companies such as Dexcom, Abbott, and Medtronic use sensor technologies that do not involve microneedles, Nutromics and Biolinq already offer a new type of sensor device based on microneedles with lengths up to 1 mm [31]. The main types of microneedles used in modern medical devices are shown in Figure 4A. The microneedle concept was proposed in the 1970s, but it was only realized in the late 1990s due to the development of microelectronic technologies; since then, the manufacturing of microneedles has been constantly improving [32]. Now, manufacturers pay attention to both the needle design (size, shape (Figure 4B), needle radius, tip angle, needle density, etc.) and the material from which it is made. During the testing process, it was found that the shorter the needles, the less painful they are when inserted, so needles are currently made up to 700 microns long, so that a balance is maintained between less tissue damage and the possibility of obtaining a sample of the analyzed liquid [33,34].

As for materials, microneedles are currently made from biocompatible materials such as metal [35], silicone [36], graphite [37], and various polymer materials, both artificial and natural [38,39]. Each type of needle has its advantages and disadvantages. For example, metal needles have high mechanical strength, dimensional accuracy and reproducibility in mass production, and are biocompatible, and some metals for their manufacture (for example, titanium) are hypoallergenic [35,40,41]. At the same time, some metals are not biocompatible, are susceptible to corrosion, are not biodegradable, and often require additional processing after manufacture; in addition, the cost of producing metal microneedles is quite high [35], so new and old production methods are constantly being developed and improved [42,43]. The classification of microneedle production methods currently used is shown in Figure 4C.

Hydrogel needles, which appeared in 2012 [47], have high biocompatibility and swelling ability, are quite easy to modify during fabrication, and are often made from available raw materials [48]. At the same time, this type of needle is characterized by low mechanical strength, and in order to increase it, manufacturers have to change the aspect ratio of the needle, its structure, or use the functionalization of materials from which microneedles are made.

In order to avoid infection when the needle is inserted in the body, each type of needle needs to choose its own type of sterilization; for example, metal needles can be sterilized using high temperature, but hydrogel needles can collapse under such conditions, so gamma radiation sterilization is more suitable for this type of needle. Sterilization of a fully assembled biosensor (with a bioreceptor layer applied) is complicated by the fact that high temperatures or toxic gases cannot be used, as this can lead to irreversible denaturation of the enzyme [49], so work in this direction is still ongoing. Since biosensor development is carried out in many laboratories, and there is a wide variety in both enzyme immobilization procedures and the materials used. There is currently no general recommendation for biosensor sterilization; therefore, such a procedure requires special attention and should be a necessary part of the process of forming a fully finished device.

In addition, during prolonged monitoring, microneedle fouling may occur [50,51]. Interstitial fluid components may settle on the surface of the needles, leading to a decrease in the sensitivity of the analysis and the intensity of the sensor signal; in addition, this may lead to false signals and, in general, to the inability to determine the concentration of the desired compound. Therefore, the microneedles have to be coated with anti-fouling coatings that prevent or reduce the non-specific adsorption of proteins and other blood components or ISF. Most often, these are biocompatible polymers, which, in addition to anti-fouling functions, can increase the noise immunity of the sensor, improve the stability of the sensitive layer, or even increase the selectivity of detection by blocking interfering substances [52,53,54]. Polyethylene glycol is often used as a crosslinking agent [55]. Its structure creates a hydrated layer around the microneedle that repels proteins and other large molecules, preventing contamination of the electrode, while the hydrophilicity of the coating can be adjusted [56,57]. Another option for reducing non-specific sorption is the use of nanoparticles, which can also improve the conductivity, sensitivity, and stability of the electrode [58,59,60]. But uniform standards have not yet been developed.

**Figure 5:**
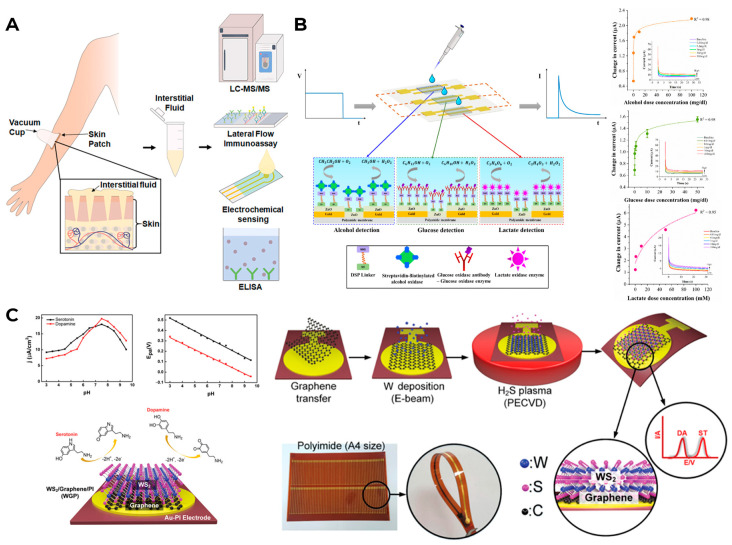
Approaches to the determination of several metabolites simultaneously: (**A**) Extraction of ISF microsamples with vacuum-assisted skin patch and shipping them to the laboratory for inpatient monitoring. Reproduced with permission: copyright 2024, Elsevier [62]. (**B**) A non-faradaic biosensor with three working electrodes for the simultaneous detection of alcohol, glucose, and lactate utilizing low volumes of sweat. Reproduced with permission: copyright 2019, MDPI [63]. (**C**) biosensor based on the WS2/graphene heterostructure on polyimide (WGP) platform for the concurrent and selective determination of dopamine and serotonin, where the signals differ due to a potential gap of 188 mV. Reproduced with permission: copyright 2021, Wiley [64].

In the future, such coatings may act as separators of sensitive layers, when one device can detect several metabolites at once, and the determination of one will not interfere with the determination of the other compound(s). So far, there are several options for simultaneous testing of various metabolites of biological fluids. On the one hand, it is possible to collect samples by the patients themselves and ship them to the laboratory, where, using various reagents, the inpatient analysis of the sent samples for various metabolites will be carried out (Figure 5A). Or, wearable devices can be created to analyze multiple compounds at once. In this case, it is possible to apply a separate layer on different needles that is sensitive to a specific compound (Figure 5B), or by varying the level of the applied potential, you can receive a signal for different connections from one electrode (Figure 5C). However, currently, there are practically no effective models of devices on the market for monitoring several metabolites simultaneously. Even cutting-edge manufacturers such as Biolinq, which plans to create a microneedle array-based sensor for measuring several analytes (glucose, lactate, and ketones), have not yet received approval to release such devices to the wider market (the initial FDA clearance for Biolinq Shine only covers glucose monitoring) [61].

Currently, the toxicity of nanomaterials and some polymers has not been fully established. So far, this is scattered information from scientific articles from various laboratories, where attempts to study the biocompatibility of nanomaterials and polymers are associated with their positive effects on the electrochemical parameters of the device as a whole, and the toxic effect is not studied separately.

**Figure 6:**
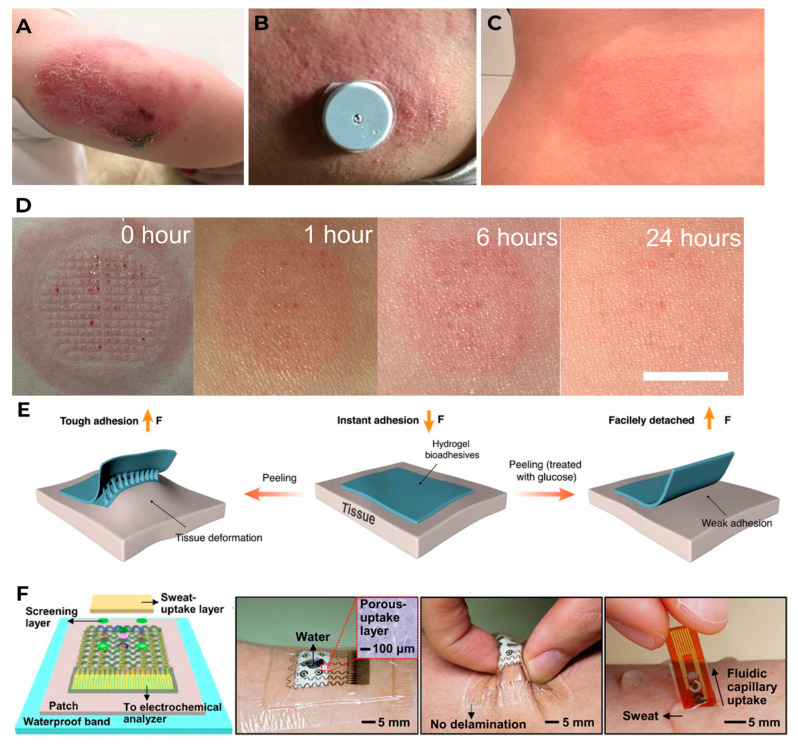
Prolonged wearing of medical devices on the skin (**A**–**C**): contact dermatitis caused by the Medtronic Guardian 3 (**A**) (reproduced with permission: copyright 2023, Oxford University Press [74]), Freestyle Libre (**B**) (reproduced with permission: copyright 2021, Wiley [75]), and Omnipod insulin pump (**C**) (reproduced with permission: copyright 2020, Wiley-Blackwell [76]); (**D**) recovery process of the skin over time after microneedle patch (reproduced with permission: copyright 2023, AAAS [77]); (**E**) glucose trigger-activated hydrogel adhesive that loses adhesion after wetting (reproduced with permission: copyright 2024, Nature Publishing [78]); (**F**) wearable waterproof biosensor patch with a sweat-uptake layer and a waterproof band (reproduced with permission: copyright 2017, AAAS [79]).

Generally, microneedles or a microneedle platform should be fixed to the surface of the skin for long-term wear. This is usually done with special patches. Researchers are faced with the task of minimizing skin irritation when wearing such devices. There may be such phenomena as itching, redness of the skin, burning, and even pain (Figure 6). The frequency of reported skin complications is quite low [65]; nevertheless, studies of such complications are constantly underway [66,67,68], and to prevent such phenomena, patches made of biocompatible materials are being developed to ensure more comfortable sensor wearing. To create patches, manufacturers use flexible printed circuit board technologies and a skin-friendly adhesive [69].

Electronic devices for biosensor signal processing, a battery, devices for wireless network connection, etc., are attached to the base, usually made with polyethylene terephthalate (PET). Polyimide (PI), polydimethylsiloxane (PDMS), polyester [70], and poly(ethylene glycol) diacrylate (PEGDA) [71] are also used as a substrate or base for placing such devices. Such a base should not only minimize discomfort when wearing but should also be strong and flexible enough to withstand continuous body movements. Moreover, since the stratum corneum of the skin is constantly being renewed, any materials will have a limited service life, and its increase with constant exposure to the external environment (water, heat, sunlight, etc.) is not a trivial task for researchers [72]. The market for electronic skin patches is projected to grow to more than $27 billion by 2033 [73].

A reasonable combination of hydrophilic and hydrophobic properties of polymers will eventually make it possible to create a patch with minimal impact on the body while maintaining the necessary functions to stay on the surface of the skin, providing minimal discomfort to the wearer. For example, in [80], the combination of polymers (flexible TPE, acrylate adhesives, polyester film, nitrocellulose, and microglass) on the surface of cellulose papers for sweat analysis allowed the patch to perform several functions at once: (i) quickly absorb sweat into the system, (ii) collect and store sweat without leakage, (iii) maintain the concentration of biomolecules and chemically unchanged sweat biomarkers, (iv) increase the volume of collected sweat to 120 µL, and (v) sequential storage of sweat produced at various time points during human testing.

The use of hydrogels such as polyvinyl alcohol (PVA) and gelatin, which have self-healing, elastic, and conductive properties [81]; a silk fibroin–polyacrylamide [82] with strong, customizable, and durable adhesive properties; and agarose, with high biocompatibility and elasticity [83], as well as their additional modification, allows such patches to increase adhesion to dry skin and maintain it with increasing humidity while using various mechanical loads, ensuring tight contact of the sensor with the skin [72].

Fixing the device to the human body is related not only to the patch material itself but also to the adhesive that can be used to fix the device to the skin for several days or weeks [84], as well as the adhesive components that are used to connect various parts of the sensor and can migrate into the patch [66]. As a rule, such adhesives may contain acrylates [85,86], rosin and its derivatives [87], and many other compounds, the presence of which can lead to allergic contact dermatitis. The most detailed analysis of possible allergens found in patches and adhesives and their effect on the development of dermatitis are presented in the studies [88,89]. The authors note that there are currently three strategies for preventing contact dermatitis when using WMDs: topical application of corticosteroids, application of barrier sprays, and placing an additional adhesive patch between the skin and the adhesive patch of the device. However, none of this eliminates the root cause of dermatitis, and these solutions can also reduce the accuracy of the device’s measurements. In fact, there is still no developed precaution to prevent skin problems. In addition, manufacturers are constantly trying to improve their products and change the composition of ingredients, which often leads to an even greater increase in the number of potential allergens [90]. The problem with identifying such allergens is complicated by the insufficient availability of information from the manufacturer, since the materials and methods used in the devices are covered by relevant patents and are not publicly available. Currently, many patients are forced to use additional medications to alleviate symptoms or, ideally, completely prevent the development of dermatitis [91]; however, this area requires further investments by manufacturers in order to be able to identify possible allergens in advance and avoid their use when creating new devices [92]. For a long time, switching to wearable tattoo-based sensors was seen as an alternative solution [93], but most of these developments never made it past the lab stage. This is mainly due to limitations in the adhesion of conductive tracks, biocompatibility due to the use of nanomaterials, the need for additional devices for reading, processing, and transmitting signals, as well as difficulties in certification and regulatory requirements [94].

All these manipulations are necessary to create a device that will firmly adhere to a human body in any conditions, especially for people having an active lifestyle, both when visiting swimming pools, saunas, baths, etc., and under various forms of physical exertion, while ensuring high-precision registration of the analyzed compound.

Another point is related to the need to miniaturize the device as a whole so that the user does not feel the “weight of wearing it”. Therefore, technologies for the development of auxiliary microelectronics with a high level of accuracy and reliability, reduced energy consumption, and high signal resolution are an important part of the development of such devices [95]. The key components are sensors for detecting the biosensor signal, power supplies, and wireless data transfer modules, as well as microcontrollers and processors for real-time signal processing. An in-depth overview of the development of power sources and energy storage systems for biosensors is presented in [96].

**Figure 7:**
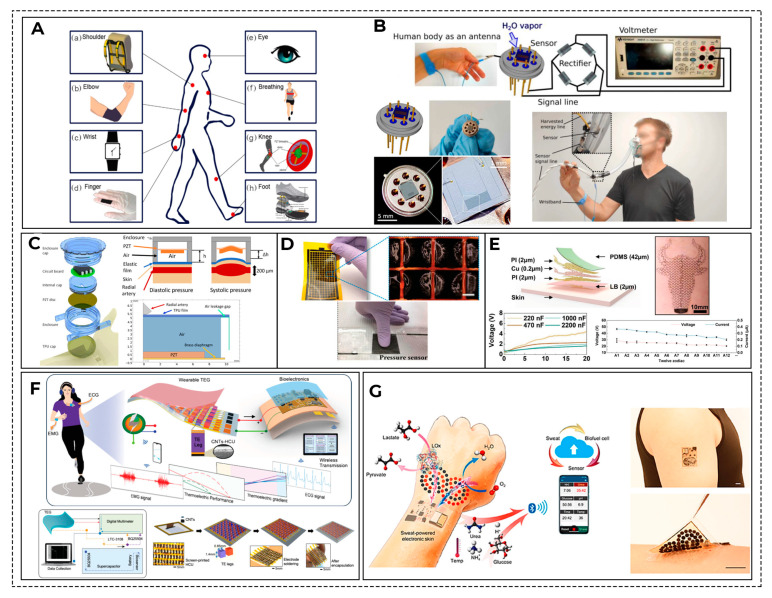
Types of power suppliers for wearable medical devices: (**A**) Several methods of extracting piezoelectric energy from various points on the person’s body. Reproduced with permission: copyright 2021, Wiley [106]. (**B**) Self-powered breath monitor enabled by electromagnetic harvesting. Reproduced with permission: copyright 2023, MDPI [107]. (**C**) 3D-printed piezoelectronic energy harvesting platform for wearable biosensors. Reproduced with permission: Copyright 2024, MDPI [108]. (**D**) A flexible high-voltage microsupercapacitor (MSC) with a planar in-series architecture for the first time based on laser-induced grapheme. Reproduced with permission: copyright 2018, American Chemical Society [109]. (**E**) Tattoo-like triboelectric generator. Reproduced with permission: copyright 2023, Wiley [110]. (**F**) Schematic illustration of a thermoelectric flexible system for wireless portable monitoring of physiological signals. Reproduced with permission: copyright 2024, American Chemical Society [101]. (**G**) Schematic of a battery-free, biofuel-powered e-skin that efficiently harvests energy from the human body, performs multiplexed biosensing, and wirelessly transmits data to a mobile user interface through Bluetooth. Reproduced with permission: copyright 2020, AAAS [111].

Moore’s Law (the density of transistors in integrated circuits doubles approximately every two years) is not applicable to batteries, so battery technologies remain a bottleneck for the development of wearable biosensors [97,98]. In addition, the batteries’ design should ensure that they can be recycled, since they contain a significant amount of valuable resources [99]. Usually, wearable devices are powered by disposable batteries, most often lithium [100]. Currently, the main efforts of manufacturers are not aimed at reducing the physical size of batteries but at increasing its energy intensity in order to increase the device service life. However, reducing the battery size is critically important to increase the comfort of using any portable medical device by the patient. Research is underway on alternative and additional energy sources, such as the conversion of body heat into electricity (thermoelectric generators) [101], the capture of energy from mechanical vibrations, such as body pulse, to power a sensor (triboelectric nanogenerators) [102], and the use of body glucose to generate energy (biofuel cells) [103]. For example, previous research [104,105] presents self-charging supercapacitors that use electrons generated when enzymes oxidize glucose as a power source. But so far, such devices are only laboratory models. Some developments in this field are shown in Figure 7B–G.

With regard to the development of wireless energy and data transmission technologies, developers using the principles of electromagnetic induction, resonance communication, or ultrasonic transmission eliminate the need for physical connections and offer safe, efficient, and reliable ways to power and communicate with electronic systems [112]. In this area, however, it is necessary to unify globally the frequencies, rules, and standards used for wireless communication of medical devices in connection with the cross-border mobility of patients using wireless systems. And although it is believed that the wireless wearable device market is still at an early stage of development, this area is ready for future growth, and according to experts, the market for wearable biosensors will reach $65,400.2 million by 2033 [113].

The development of these technologies has made it possible to market fairly lightweight sensors that reduce wearing discomfort; for example, the FreeStyle Libre continuous glucose monitoring sensor weighs 65 g, while the weight of the sensor itself, worn on the body, is 5 g [114], the Dexcom G6 sensor is 28.35 g [115], Dexcom G7 is 3.3 g approximately for the all-in-one sensor and transmitter [116], the TOSHIKO CGM Sensor is 2.16 g [117], the Lingo Biosensor is 0.21 pounds (approximately 95g) [118], and the AiDEX™ Continuous Glucose Monitoring System is 5.5 g [119]. Further development of micro- and nanotechnology is likely to further reduce the weight and size of such devices.

## 3. Sensor Measurement Error

In addition to the comfortable collection of samples for analysis and comfortable wearing of the device, the characteristics of the biosensor itself, which directly analyzes the metabolite level in the body, are important. Here, it is necessary that the device determines the concentration of the analyzed compound as accurately as possible, since this is important not only for monitoring its level in the body but also, in case of deviation from the physiological norm, for timely assistance and normalization of the human condition.

Let us take a look at what could be causing measurement errors in currently developed wearable sensors.

**Figure 8:**
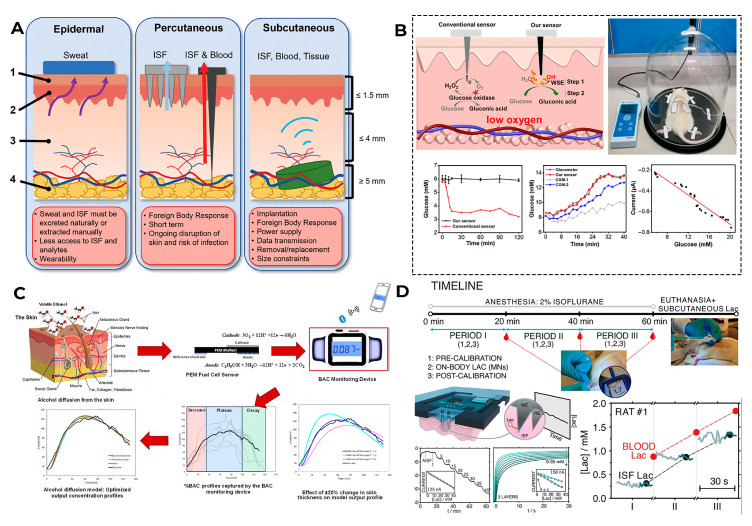
Possible problems with the measurement of metabolites in ISF. (**A**) Classification of skin-based biosensors: (1) stratum corneum, (2) epidermis, (3) dermis, and (4) subcutaneous tissue. Reproduced with permission: copyright 2024, Wiley [95]. (**B**) Difference in the accuracy of glucose measurement in ISF using enzymatic and non-enzymatic biosensors under hypoxic conditions. Reproduced with permission: copyright 2023, MDPI [120]. (**C**) Kinetics of alcohol detection using a non-invasive wearable alcohol monitoring device and the effect of skin epidermis thickness and diffusion process time on alcohol concentration dynamics. Reproduced with permission: copyright 2024, MDPI [125]. (**D**) Comparison of blood and ISF lactate concentrations in rats measured with a microneedle-based sensing system. Reproduced with permission: copyright 2024, American Chemical Society [131].

First, most minimally invasive devices currently being developed measure the concentration of the analyzed compound not in blood but in ISF (the results of some of the studied problems related to ISF measurement are shown in Figure 8). As for measuring, for example, glucose, its levels in these liquids do not always match perfectly, especially when rapid changes occur in the concentration: it takes about 5–15 min for glucose from their blood vessels to enter the ISF [120,121]. Also, when taking insulin, it was found that fast-acting insulin begins to lower glucose levels after about 15 min, reaching a peak effect after about an hour, and the readings of modern sensors, although reflecting a tendency to decrease glucose levels within a few minutes after injection, still lag behind the actual blood glucose level [122,123]. Therefore, in such situations, the sensor readings will show an incorrect glucose concentration, a fact that must be taken into account for taking the appropriate medication.

As for sensors for determining alcohol content, its concentration in blood and ISF correlate strongly [124], as well as in blood and sweat under certain conditions [125], which contributed to the development of wearable sensor platforms for determining alcohol in ISF [126] and in sweat [127,128].

When analyzing lactate, it should be taken into account that in some situations, for example, in pathological conditions (sepsis), lactate levels in ISF can outpace its blood levels [129,130], making ISF a potentially earlier marker of problems at the tissue level. However, individual indicators (for example, obesity, physical activity intensity, etc.) may affect the accuracy of measuring ISF levels compared to blood values [131,132]. Therefore, when developing wearable minimally invasive lactate detection devices, it is necessary to take into account the dynamics of lactate in various human body fluids and monitor clear temporal patterns of changes in the metabolite levels of them, possibly using machine learning to model interindividual physiological differences in lactate kinetics in blood, ISF lactate, and sweat [133,134].

**Figure 9:**
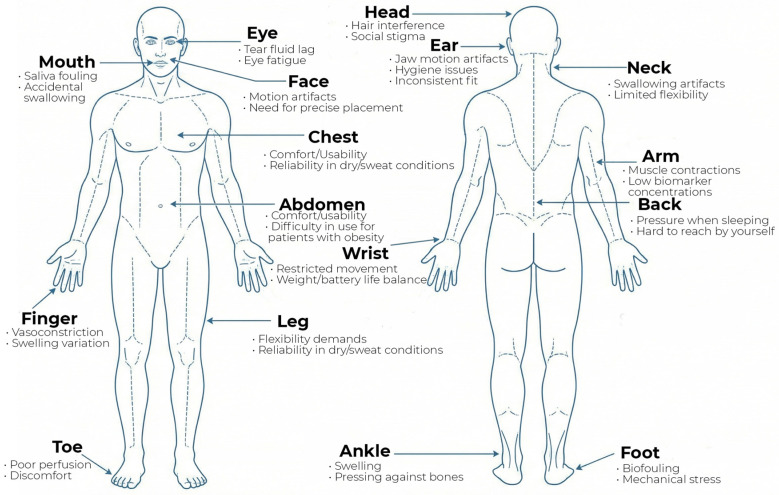
Installation locations of wearable medical devices on the body and typical difficulties associated with them.

The second important factor leading to measurement errors is the sensor location on the body. As a rule, the instructions for the device pay attention to this. This is due to the fact that different parts of the body have different blood flow, tissue structure, and activity, which can affect the sensor readings [135]. Figure 9 shows some of the difficulties of using and receiving data from wearable devices, depending on where they are installed.

For example, for healthy people, it has been shown that differences in glucose readings can occur even between two arms. The study [136] assessed the average difference in glucose levels between the right and left arms and the effect of unilateral exercise on this difference, and it was shown that the average glucose level in the right arm was significantly higher than in the left arm by 3.7 mg/dL (*p* < 0.001), and this result was not affected by diet or exercise for the arm. Glucose levels were in the euglycemic range 75.2% of the time in the right arm and 67.5% of the time in the left arm (*p* < 0.001).

Among the glucose sensors currently available on the market, for example, Dexcom sensors (such as the G6 and G7) are approved for use on the abdomen and the back of the upper arm, with the G7 specifically recommended for arm placement for optimal performance (and for children ages 2–6, the upper buttocks may be used). In contrast, Abbott’s FreeStyle Libre sensors are approved solely for application on the back of the upper arm, while Medtronic’s Guardian Connect sensors are approved for use either on the abdomen or the back of the upper arm, provided there is an adequate amount of subcutaneous fat [137]. Another study [138] demonstrated that FreeStyle Libre sensors can also function when installed on the upper thigh, but when installed in the abdominal area, they perform unacceptably poorly.

The optimal location for placing a continuous glucose monitor sensor is the abdomen, specifically at least 2 inches away from the navel and insulin injection sites, as it provides the most stable and accurate readings [139]. Experiments have shown that the accuracy of a Dexcom G4 Platinum CGM sensor placed on the upper arm was not different from the accuracy of the sensor placed on the abdomen in adults with type 1 diabetes [140]. At the same time, for healthy people, tests have shown that at rest, traditional placement of CGM sensors on the arm may best reflect blood glucose; however, during cycling, placement on the leg may offer greater insight to working muscle glucose concentrations, and this is likely due to greater blood flow rather than muscle contraction [135].

It is worth noting that incorrect sensor installation or displacement, for example, during sleep or exercise, can also lead to measurement errors, from which any user is not immune. So, back in 2013, a systematic study was conducted on the indications of percutaneous CGM while lying on devices (for example, in various positions during sleep) [141]. It was shown that individual sensors periodically showed abnormal glucose readings (deviation from the median by more than 25 mg/dL) and that these abnormal readings were directly related to the fact that the subjects were lying on the sensors and exerting excessive pressure on them. Most of these abnormal CGM readings related to sleep position represented a sudden decrease in the reported glucose values, so the authors suggested that they were due to a local decrease in blood flow caused by tissue compression. CGM sensors can be easily moved to other places on the body that are not exposed to such risks. However, if we are talking about other sensors, for example, wearable sensors that will detect concentrations of metabolites in certain organs and which should be located in a strictly defined place in the body, then manufacturers need to consider other strategies to overcome this problem.

Problems with measurement accuracy are also related to the individual variability of blood, ISF, and other body fluids in which the analyte content is measured, both within the subject and between patients. Differences in WMD readings may be due to heart rate, respiratory rate, skin temperature, physical activity, time of food intake and its quality and quantity, metabolic rate during the day, the presence of inflammatory processes in the body, stress, etc. [142,143,144,145]. Thus, in some cases, personalized device calibration is needed for each person, which is not yet feasible with the available technologies and economically unprofitable. Nevertheless, attempts to develop approaches for personalized calibration of such devices are underway, and they are related to machine learning, neural networks, and optimization of various device parameters, leading to a reduction in the error of measuring and predicting glucose levels [146]. This is especially important for hybrid devices based on insulin pumps, which must monitor possible moments of hyperglycemia and hypoglycemia in order to take the necessary therapeutic measures in time. For example, modern glucose meters for non-invasive continuous glucose monitoring exhibit extremely large errors in measuring low blood glucose concentrations (<1%) using a non-invasive sensor due to variations in one patient and between them [147]. Therefore, the analysis of data received from patients is very important and helps to track not just individual glucose levels but the dynamics of individual changes/fluctuations in a particular person’s body [148,149], compares different monitoring systems according to their characteristics [150,151], and helps manufacturers move forward.

Another important factor is the effect of body temperature at the sensor installation location on the readings of wearable medical devices. Sixty-five years ago, Wilhelm Graf [152] reported differences in the temperature of internal organs of about 0.2 °C. In different diseases, this difference can be even more significant. For example, in neurological injuries (traumatic brain injury, subarachnoid hemorrhage, and stroke), the temperature of the brain tissue may differ from the temperature of the rest of the body and in both directions [153]. One of the most important tasks of modern medical sensors is to eliminate the influence of temperature fluctuations on the readings of the device. Twenty years ago, traditional glucose meters showed that measurements on the cold skin of the finger and forearm show significantly lower concentrations of glucose levels compared to measurements under normal conditions, and the measurement time increases dramatically [154]. The serious influence of ambient temperatures on the measurement accuracy of such sensors has also been demonstrated [155]. With the transition to measurements using wearable CGM devices, this problem should have disappeared; however, the researchers still note that the choice of the sensor’s location on the human body can still significantly affect its readings, including due to fluctuations in blood flow in the skin and differences in local body temperatures [33,156]. This problem is especially evident in non-invasive sensors, which are based on spectral analysis methods [157]. Therefore, one of the key areas of research is currently the development of temperature-insensitive sensor compositions. One way to achieve this is to develop temperature-insensitive conductive polymers that exhibit reduced permeability to metabolites while increasing the O_2_ permeability during use, which allows sensor signals to be stabilized over a certain temperature range [158]. Another option is to install additional local sensors for temperature correction [159].

Finally, most wearable sensors are enzyme-based biosensors, so their readings can be greatly influenced by various metabolites in the blood, including medications taken by the patient [160,161]. For example, some over-the-counter painkillers, such as acetaminophen (Paracetamol and Tylenol), can affect the operation of continuous glucose monitoring systems [162,163,164]. High doses of acetaminophen can lead to falsely inflated readings due to the formation of acetaminophen glucuronide in the interstitial fluid, i.e., the drug itself does not affect the actual glucose level but can oxidize on the sensor and cause false high readings of glucose meters, especially in amperometric biosensors based on measurements of hydrogen peroxide [165]. The Dexcom G6 sensor has a reduced sensitivity to acetaminophen; however, taking a dose exceeding the maximum may distort the readings. Alternatively, patients are encouraged to use alternative painkillers that do not affect the continuous glucose meter readings, such as nonsteroidal anti-inflammatory drugs [166].

At the same time, some steroids can increase blood glucose levels [167], reducing the body’s sensitivity to insulin, so when taking them, you need to evaluate the glucose meter readings in a different way and be sure to consult a doctor. Some medications for the treatment of high blood pressure (trademarks Qbrelis, Zestril, and Prinivil) relax blood vessels, increasing blood flow and oxygen to the heart, and, for example, lisinopril, especially in the first couple of weeks of use, can lower blood glucose levels [168].

Dexcom G6 indications may be affected by hydroxyurea [169], which is an antitumor drug used primarily in the chemotherapy of certain forms of cancer, as well as for the treatment of sickle cell anemia, which may lead to an overestimation of the actual glucose levels. The operation of Medtronic’s continuous glucose monitoring systems (Guardian 4 CGM) is also hindered by the intake of hydroxyurea [160]. For Abbott’s FreeStyle Libre sensors, excessive intake of vitamin C (ascorbic acid) can affect sensor readings, especially in doses above 500 mg per day, and low blood sugar levels may not be noticeable against this background [170]. Similarly, aspirin or other drugs containing salicylate may cause the sensor to register lower glucose levels than the true values [171]. The review [172] provides the most comprehensive list of chemicals that have been identified as possible interference with continuous glucose monitoring systems currently or previously commercially available.

**Figure 10:**
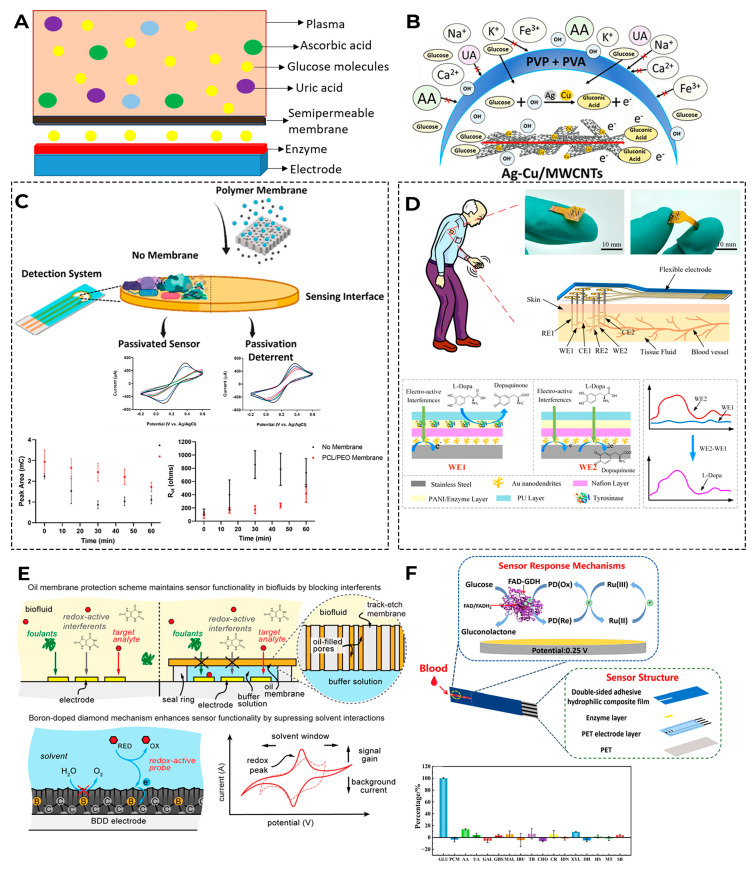
Control of interfering substances in glucose sensors. (**A**) Basic working principle of semipermeable membranes. Reproduced with permission: copyright 2016, MDPI [180]. (**B**) A PVP/PVA hydrogel coating that protects Ag–Cu/MWCNT nanocomposites that are used for glucose determination. Reproduced with permission: copyright 2025, American Chemical Society [181]. (**C**) Usage of PCL/PEO polymer membrane that prevents biofouling of microelectrodes in wearable sensors. Reproduced with permission: copyright 2023, MDPI [182]. (**D**) A minimally invasive L-Dopa biosensor based on a flexible differential microneedle array, where one working electrode responded to L-Dopa and interfering substances, while the other working electrode only responded to electroactive interferences. Reproduced with permission: copyright 2022, MDPI [183]. (**E**) Combined oil–membrane protection and boron-doped diamond (BDD) approach for mitigating biofluid effects. Reproduced with permission: copyright 2021, MDPI [184]. (**F**) A glucose sensor based on the use of the dual redox mediator 1.10-Phenanthroline-5.6-dione (PD)/Ru(III) and its signals for commonly used drugs, anticoagulants, and food-derived substances. Reproduced with permission: copyright 2025, MDPI [177].

Manufacturers are seeking different solutions of such situations: for example, they can change the measuring voltage at which other compounds will not oxidize on the electrode [173,174,175]; they can use diffusion membranes that block access of other compounds to the sensitive surface of the sensor [176]; and they can use a mediator that will interact only with the enzyme used [177] or apply additional enzymes or oxidants that will break down interfering compounds [178]; in addition, they can use mathematical methods of signal processing and machine learning [179]. Figure 10 shows some schematics for glucose biosensors, which minimize the interfering compounds on the sensor reading’s effect.

Of course, sensor manufacturers have to constantly monitor the emergence of new therapeutic drugs and their impact on sensor performance. At the same time, the results of their research should be available both to doctors to make the right decision about the patient’s treatment, and to patients to exclude or limit appropriate foods from their diet (regarding vitamin C). However, currently, in case of doubt, manufacturers still recommend using traditional glucose meters with blood measurement from a finger.

The solution to this problem is gradually getting closer with the study of new methods of signal generation by biosensors. Currently, existing biosensors are usually divided into several generations. First-generation systems (such as those used in commercially available Dexcom STS and G7 devices) are based on measuring oxygen and hydrogen peroxide produced during the glucose oxidation reaction under the catalytic action of the enzyme glucose oxidase, but the measurement results are strongly influenced by ambient oxygen and reducing agents such as ascorbic acid and uric acid in the blood, which can negatively affect the accuracy of glucose levels [185]. The second-generation systems are based on measuring the current generated when electrons are transferred from the active enzyme center to the electrode surface using appropriate mediators. The FreeStyle Libre sensors from Abbott, SiAmbulatory sensors from Shenzhen SiBionics, as well as AiDEX^®^ and AiDEX^®^X sensors from MicroTech Medical are based on this principle. These sensors are considered potentially toxic due to the potential for leaching of mediators and instability during prolonged use [186]. In third-generation sensors, it was possible to get rid of mediators by achieving direct transfer of electrons from the active enzyme center to the electrode without additional mediators [187]. So far, such sensors are at the stage of laboratory research [188]. Direct electron transfer can be achieved using conductive polymers [189], nanomaterials [190], or by changing the conformation of the enzyme using chemical compounds [191]. Such sensors are sensitive to ambient temperature, humidity, etc.; in addition, although the use of nanomaterials gives significant advantages to devices based on them, their effect on various organisms, and especially humans, has not been fully studied [192,193].

The fourth-generation sensors are non-enzymatic and are based on direct electrochemical oxidation of a metabolite molecule on the electrode surface at a certain potential and conditions [194]. For this purpose, electrodes made of catalytic materials are used: metals and their oxides with several degrees of oxidation, conductive polymers, and nanomaterials [195,196]. This minimizes the effect of interfering substances because it is possible to select measurement conditions in which only the compound being determined is oxidized. But problems with the stability of such electrodes are still in the process of being solved. And besides, the use of nanomaterials raises serious concerns from a medical point of view, as we said above.

It is worth noting that incorrect measurement results may occur immediately after installing the sensor, as it takes a certain period of time to wet the sensor and stabilize it, since the biosensor is dry during transportation and storage. Typically, the sensor’s “warm-up” time can range from 30 min to 2 h, depending on the manufacturer. For example, Dexcom G7 glucose sensors sold have a shorter warm-up period of about 30 min, FreeStyle Libre sensors often take about an hour, and, for example, the Medtronic Guardian device requires a two-hour warm-up period. Perhaps this problem will be able to be solved in the fourth-generation sensors, but for biosensors, most likely, it will not be possible to completely avoid some adjustment time after startup.

## 4. Problems of Real-World Biosensor Use

In addition to the problems directly related to the accuracy and reproducibility of the readings obtained by the biosensor working electrode, there are many other important aspects for the effective operation of wearable medical devices. First of all, this concerns the features of the devices’ operation in conditions other than ideal. Biological fluids such as sweat, tears, saliva, blood, urine, gastric juice, etc., are a rich source of signals for active wearable biochemical sensors. The measurement accuracy over the entire concentration range and the effects of interfering compounds are easy to predict in laboratory tests, but a number of problems can occur only with real-world device operation, depending on the biological object they are aimed at measuring the metabolite concentration.

So, there are different opinions about what the time of use of one wearable/minimally invasive/invasive medical device should be. Most CGM devices are currently designed for ~2 weeks of use before the sensor is replaced, which is due to both a drop in the signal of the recognition element over time and numerous medical factors. However, there are also devices on the market that provide measurement for 365 days without the need to replace any elements: Eversense 365 CGM. Unlike the majority of other electrochemical CGM devices, this is an optical sensor that consists of an array of four glucose and oxidation sensing areas, which mitigates the effects of both local oxidation of the glucose indicator molecule and immune-mediated degradation. According to a year-long study of the effectiveness of these devices [197], 90 percent of the sensors installed in 110 diabetic patients worked for 365 days, with the overall mean absolute relative difference of 8.8%. However, such impressive results can only be achieved with weekly calibration of the device, which allows for timely correction of the sensor readings. Moreover, both the installation and calibration of the sensors were carried out by trained medical staff in a specialized medical institution. Obviously, not all wearable and even more invasive biosensors can be calibrated using an external device, and even more so, doing it so often is too much of a burden on real patients.

The detection of metabolites in various biological fluids allows for non-invasive analysis, which is less traumatic for humans, such as saliva analysis. Saliva contains molecular information that can reflect a person’s health status and can be used as a tool for identifying biomarkers of oral diseases [198] and other diseases, even neurodegenerative and mental disorders [199], as well as measuring air pollution associated with road traffic [200]. It has been shown that human saliva and serum metabolomes are comparable in chemical composition but exhibit significant differences in the concentrations of common metabolites [201]. Although knowledge about saliva and its homeostasis has expanded over the past two decades and data have been collected on compounds present in saliva that are related to the genome, epigenome, transcriptome, proteome, metabolome, and microbiome of the subject, at the same time, the intense metabolism in the oral cavity prevents very strong correlations between the concentrations of various compounds in serum and saliva. Therefore, saliva analysis still complements and expands on blood analysis data but cannot completely replace it.

Long-term wear of sensors based on other biological fluids, such as tears, is also problematic. The NovioSense Glucose Sensor is the very first sensor reported for continuous measuring of glucose levels in the basal tear fluid, and the authors claimed it correlates glucose in blood with clinical viability [202]. However, many researchers note that reduced sample volume, lower glucose levels, and problems with tear evaporation create problems with correlation with blood glucose levels [203]. Different types of tears (reflex, emotional, or mechanically induced) may have different chemical compositions [204], and wearing contact lenses with built-in biosensors [205] for a long time is considered undesirable. The limited volume of non-stimulated tear film is a significant obstacle to the detection of other biomarkers in tear fluid [206], but researchers are proposing solutions to this problem, for example, using aptamer-based affinity nanobiosensors [207].

As for urine, urine metabolites and blood serum metabolites generally correlate weakly [208,209] because these fluids reflect different biological processes. Serum is associated with systemic circulation and homeostasis, while urine reflects cumulative excretion and is associated with metabolic products and kidney function. Certain conditions, such as certain gastrointestinal disorders (Crohn’s disease, ulcerative colitis, celiac disease, protein-losing enteropathy, etc.), can cause characteristic changes in serum but do not cause dramatic changes in urine composition [210,211]. Nevertheless, the numerous correlations identified between urine metabolites and various clinical and biochemical indicators suggest that urine metabolites may be of general importance as biomarkers of health and disease. This is particularly relevant for the detection of diseases in children in order to reduce the painfulness of sample collection for analysis.

**Figure 11:**
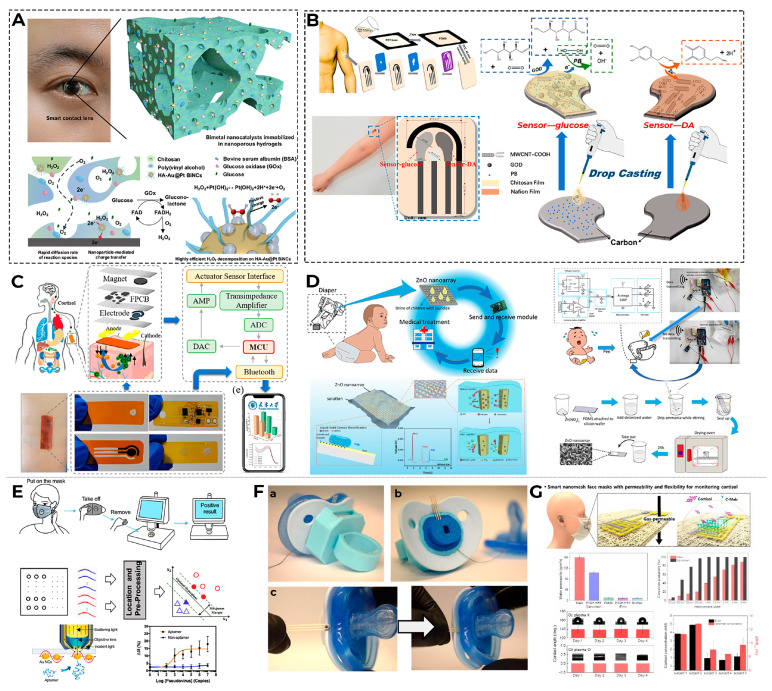
Schemes for measuring metabolites in various physiological fluids (tears—**A**; sweat—**B**; interstitial fluid—**C**; urine—**D**; saliva—**E**–**G**). (**A**) Smart contact lenses for continuous glucose monitoring based on bimetallic nanocatalysts immobilized in nanoporous hydrogels of the lens. Reproduced with permission: copyright 2022, Wiley [214]. (**B**) A wearable electrochemical patch sensor for simultaneous detection of dopamine and glucose in sweat. The scheme of substrate preparation and the electrode printing process is shown; the prepared patch sensor on the recipient’s skin, as well as the modification process and the mechanism of electrochemical detection. Reproduced with permission: copyright 2023, MDPI [215]. (**C**) Non-invasive, wearable cortisol sensing system. The process of cortisol secretion by the human body, explosion diagram, photos, and the working principle of the sensing system are shown. Reproduced with permission: copyright 2025, MDPI [216]. (**D**) Self-powered wearable biosensor in a baby diaper for monitoring neonatal jaundice using ZnO nanowires for detecting bilirubin. Reproduced with permission: copyright 2022, MDPI [217]. (**E**) Wearable biosensor for long-term saliva collection and self-detection of SARS-CoV-2. Reproduced with permission: copyright 2023, MDPI [218]. (**F**) A fully integrated pacifier that works as a portable wireless device for non-invasive chemical monitoring of glucose levels in baby saliva (back part of the pacifier—a; electrochemical cell—b; electrode replacement—c). Reproduced with permission: copyright 2019, American Chemical Society [219]. (**G**) A flexible and permeable nanomesh-based wearable biosensor designed for bioelectronic face masks that monitor cortisol levels. Reproduced with permission: copyright 2025, American Chemical Society [220].

The most readily available biological fluid for use in wearable sensors is sweat, as sweat glands are distributed throughout the body. Therefore, biosensors that measure the level of the metabolite in sweat and are located on the skin are theoretically the most suitable for long-term use. Researchers are mostly focused on measuring glucose, lactate, electrolytes, and hormones in sweat [212]. Recent advances in materials science and flexible electronics have made it easy to embed sensors in bandages, tattoos, clothing, and accessories. However, since the analytes in sweat mainly enter sweat through capillaries, it is difficult to establish a clear and accurate relationship with blood concentrations, especially over a long period of time [213]. The degree of analyte dilution during perspiration is also affected by the rate of perspiration and the rate of analyte distribution. Another factor affecting the accuracy of the analysis is the gradual contamination of the sensor surface with salts and interfering metabolites, which are found in excess in sweat. Finally, for the elderly, sick, or persons with disabilities, whose physical condition does not allow them to perform exercises that cause sweating, sweat sensors lose their usefulness as sweating becomes less intense. The measurement schemes of metabolites in various physiological fluids are shown in Figure 11.

If we consider implantable and semi-implantable devices, then a significant problem even for miniature in situ sensors is that they must be removed after performing their function. Surgical removal causes discomfort in patients and can lead to postoperative complications. Therefore, there are two ways to solve this problem: either the maximization of the sensor’s service life (up to decades) or the biodegradability of implants and their parts for their removal through natural biological processes [221]. For example, there are biodegradable sensors for measuring blood flow [222], pressure sensors [223], as well as biodegradable displays for wearable electronics [224].

Wearable biosensors often require special storage conditions to preserve their sensory properties during transportation and before use, especially because biological components such as enzymes are inherently unstable outside of optimal storage conditions. Temperature is considered the most important factor for maintaining biosensor operability during storage and transportation. Studies of aptamer-based electrochemical sensors show that storage at −20 °C is sufficient to maintain biosensor functionality for at least six months without the need for exogenous preservatives [225]. Puggioni et al. [226] demonstrated the variations in kinetic parameters of glucose and lactate biosensors under different storage conditions. Their results also demonstrated the preservation of bioreceptor activity when stored at −20 °C, and when stored at −80 °C, even higher V_MAX_ sensor values were recorded compared to the other two groups. Similar data were obtained when testing the glutamate biosensor in the presence of glycerol and triethylene glycol as stabilizers [227]. However, the option of storing at ultra-low temperatures is practically impossible outside of laboratory conditions. An intermediate option is considered to be storing the electrodes in a refrigerator (temperature +4 °C), but according to some data, at this temperature, the decrease in biosensor activity during storage for 4 months can be up to 50% of the initial value [226]. The shelf life of most commercial CGM sensors is typically between 6 and 18 months from the date of production, depending on the manufacturer. The main reason for the decrease in sensor signals during storage is the deactivation of enzymes. Thus, a decrease in glucose oxidase activity in CGM can be influenced by several factors at once: inactivation of the active sites of the enzyme by hydrogen peroxide, a change in the conformation of the enzyme, and degradation due to certain low-molecular compounds [228]. Some of these problems are solved by choosing the optimal method of enzyme immobilization and numerous protective membranes covering the biological layer. However, even reliably immobilized enzymes require protective packaging to ensure long-term storage. Currently, the most effective methods of storage use a combination of vacuuming, freeze-drying methods [229], and humidity control using additional reagents [230]. However, manufacturers still need to pay close attention to this aspect. In particular, it is not always possible to ensure strictly controlled storage conditions during the transportation of sensors and their sale. It is possible to use specialized materials for thermal stabilization: polymers, ceramic composites, and heat-resistant alloys, which would act as thermal buffers during transportation, preventing a decrease in productivity due to temperature fluctuations.

In autonomous wearable biosensors, sensitivity and stability are often performance indicators that limit each other [231]. As a rule, devices with high sensitivity are more susceptible to changes in environmental factors, which can lead to instability of sensor signals. Therefore, an important task for the creators of wearable sensors is to find a balance between the maximum possible duration of wearing the device and its sufficient accuracy. Also, the measurement accuracy may vary depending on the measurement conditions and the special conditions of the patient. The problem is that this relationship is quite difficult to detect in the framework of laboratory studies. For example, in CGM, when assessing the effect of interfering substances on measurement accuracy, it is often impossible to obtain sufficient amounts of ISF, and one has to rely on artificial ISF with parameters close to the real one [172]. However, under such conditions, it is difficult to assess the ISF’s ability to contaminate or passivate CGM electrodes and take into account the presence of all possible substances. An additional complication is that many classical laboratory tests are performed on bare (unmodified) electrodes, where passivation effects can be most pronounced, while commercial CGM devices contain membranes designed to reduce the flow of potential pollutants from the environment into the sensor filament structure while simultaneously supporting the diffusion of intermediates from the active layer of the reagent to the electrode surface (described in Section 2 of this review). Thus, the behavior of the passivating agent in vitro may not lead to a similar effect in vivo [232].

Unresolved issues include the behavior of sensors during extreme metabolic episodes, which can lead to significant changes in the levels and overall balance of electrolytes in the body: diabetic ketoacidosis, lactic acidosis, and others characterized by a rapid and significant decrease in blood pH, which can also affect the accuracy of glucose measurement while wearing the sensor [233].

Wearing systems in vivo for days or weeks in which the sensitive part of the device is located under the skin makes these devices more susceptible to local effects, such as tissue injury aggravated by constant body movements, local immune reactions, and the risk of biological tissue contamination/tissue formation above the sensitive element, which can lead to a longer-term deterioration of the signal specific to glucose levels. The goal of the researchers is not only to create the most accurate and viable wearable medical device but also to make it the least disruptive to a patient’s full daily life. In particular, the microneedles used in most available CGM systems, although minimally invasive, are still accompanied by a risk of skin irritation and infection. CGM systems such as tooth tattoos, skin tattoos, contact lenses, and watches have been proposed to minimize these risks [234,235,236,237,238]. However, so far all of them exist only in the form of laboratory prototypes and have not reached a wide market. One possible solution to this problem is the emergence of smart textiles that combine the functions of sensors and energy sources. Clothing is something that everyone wears on a daily basis, and the integration of electrochemical biosensors into textiles simplifies the collection and transmission of health data about the person who wears them. Users of such devices can enjoy the benefits of technology without the discomfort and inconvenience associated with wearing traditional rigid electronic devices [239]. Currently, prototypes of flexible wearable textile sensors for uric acid [240], lactate [241], glucose [242], and even sensors for assessing CRB levels in order to detect inflammatory cardiovascular diseases [243] have been proposed.

We have already touched upon the need for individual tuning of biosensors that use sweat as an analyte because a potential change in the intensity of sweating in patients requires constant active calibration of the device. But the problem of calibration for a specific patient exists for any biosensor, because due to the complexity of biochemical reactions, it is not always possible to achieve an ideal match of indicators within a batch of devices directly “from the factory”. The first generations of CGM circumvented this problem by using an external standard, i.e., by comparing it with a classic glucose meter with test strips, which gives data after a fingerstick. This was not an ideal solution, as it required additional equipment for the patient and, to some extent, went against the very idea of non-invasive, non-traumatic CGM. In addition, the strips themselves do not always have sufficient accuracy [244,245]. An ideal CGM sensor should not require calibration using a fingerstick; instead, some other parameter is needed by which the dynamics of changes in glucose sensor signals can be adjusted. One possible solution for this could be to measure interstitial Na^+^ along with glucose using a subcutaneous electrochemical sensor. The concentration of Na^+^ in the interstitial fluid remains approximately constant over time [246]. Therefore, it can potentially be used as an excellent internal standard for comparing blood glucose and interstitial fluid without the need to take blood samples for calibration [247]. Another option for adjusting CGM indicators is to develop integrated analysis systems linked to pulse measuring devices to improve the accuracy of glucose measuring [248]. Modern wearable biosensors use more complex signal processing methods that go beyond simple linear calibration models. They may utilize statistical data obtained at the population level or from previous sensor usage [249], mathematical methods of high-frequency filtering and dynamic signal processing, as well as complex algorithms based on machine learning and artificial neural networks trained on individual patient data to create personalized prediction models [204]. The new algorithms allow calibration parameters to change over time, reflecting the fact that the relationship between sensor and analyte can change due to tissue inflammation, enzyme degradation, or other biological factors [250]. Another calibration strategy is to use built-in reference systems that allow the sensor to recalibrate itself without the need for any patient measurements. For example, some devices contain internal reference electrodes or calibration compounds that are periodically released to check the sensor’s operability [251].

**Figure 12:**
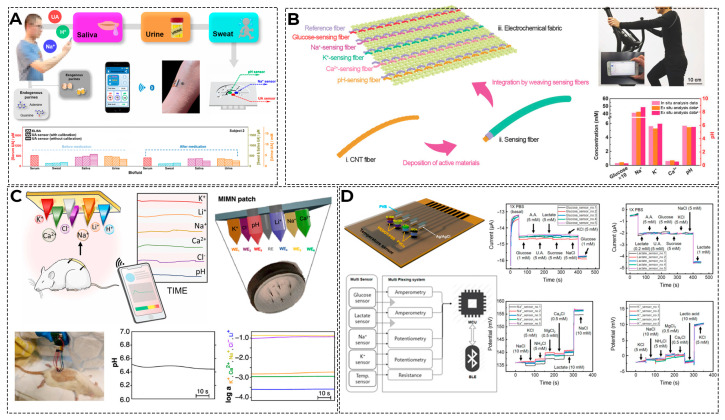
Wearable devices using multiple sensors in one. (**A**) Universal fully integrated wearable sensor arrays for simultaneous metabolite and electrolyte detection in urine, sweat, and saliva. Reproduced with permission: copyright 2022, American Chemical Society [252]. (**B**) Weaving sensing fibers into electrochemical fabric for real-time monitoring of glucose, Na^+^, K^+^, Ca^2+^, and pH. Reproduced with permission: copyright 2018, Wiley [253]. (**C**) Multi-ion transdermal monitoring system with a potentiometric microneedle-based sensor patch. Reproduced with permission: copyright 2022, American Chemical Society [254]. (**D**) Laser-induced graphene-based multiplexed biosensing system for measurement of glucose, lactate, Na^+^, K^+^, and temperature. Reproduced with permission: copyright 2024, MDPI [255].

Examples of laboratory models of devices currently being developed that are sensitive to different body parameters, allowing for more accurate identification of the main metabolite, are shown in Figure 12.

In general, simultaneous non-invasive monitoring of a wide range of biomarkers and physiological parameters is the future of wearable medical devices. Today, modern wearable biosensors are increasingly combining several measurement methods, combining electrochemical, optical, and mechanical measurements of the same or related analytes. This multiplex approach provides built-in cross-validation and allows you to dynamically adjust measurements based on auxiliary sensor data. For example, a glucose sensor can be equipped with pH, temperature, and humidity sensors that adjust the measurement results to reflect environmental changes affecting the operation of the enzymatic sensor. Such a comprehensive analysis will not only provide a more complete picture of the physiological condition of patients but also ensure active calibration and correction of the device’s response for more accurate monitoring.

## 5. Data Transmission

Data transmission from wearable biosensors is a complex multi-step process during which physiological signals are transformed into useful health information for end users and healthcare providers. If the data from the sensor element is not transmitted reliably and safely enough to the device processing them, this can lead to an erroneous diagnosis or to a lack of timely response to critical changes.

Data transmission to the end user begins with analog signals received by the working electrodes of the sensors: this can be a change in current or voltage in the case of electrochemical sensors or, for example, light adsorption in the case of optical sensors [256]. Due to the miniaturization of most wearable sensors, the signals from them are usually also quite small. In addition, they are susceptible to external interference and artifacts caused by human movements. Therefore, first of all, these useful signals need to be isolated from the background data and amplified [257]. There are many ways to optimize and amplify signals, in which amplifying circuits enhance the detected analog signal, while suppressing common-mode interference [258]. After successful signal amplification, it must be filtered from noise, and this must be done with the highest possible accuracy [259]. After such preparation, the signal must be transmitted to a processing device, and for this, it must be converted from analog to digital. For wearable biosensors, the choice of an analog-to-digital converter (ADC) significantly affects the power consumption and accuracy of the device. Sequential Approximation Register (SAR) ADCs are popular in wearable devices with limited power consumption [260], while sigma–delta ADCs offer higher resolution due to greater computing power [261]. In addition, it is possible to combine different types of converters to create systems with the most optimal performance characteristics for each specific device.

Finally, the converted digital data must be transferred from a wearable device to a certain processing center: it can be a separate chip somewhere on the sensor itself, a user’s device (smartphone) or a cloud platform. There are several wireless communication standards that meet different application needs, each with its own advantages and disadvantages in terms of range, power consumption, and data transfer rate. According to statistics, autonomous biosensors mainly use signal transmission technologies that consume energy in the range of 0.01–130 MW (RF communication, Bluetooth, Wi-Fi, Zigbee, or near-field communication (NFC)) [231].

Due to advantages such as excellent compatibility, good data security, and low power consumption, Bluetooth technology has been used in almost all portable/wearable electronic devices since its introduction. The size of Bluetooth modules used in modern autonomous wearable biosensors today does not exceed 1 cm^2^ [262], and the data transmission range is about 100 cm. Bluetooth and its variations (Bluetooth low energy) allow to use sophisticated packet loss minimization strategies that are crucial for health monitoring. A comprehensive assessment of packet loss in Android and iOS-based wearable systems has shown that a combination of various methods, including frequency reduction, data pooling, and queuing packet transmission protocols, can reduce packet loss to less than 1%, which is critically important in the medical field [263]. However, the Bluetooth module consumes a lot of energy at the time of connection [264], and its average power consumption can reach quite high values. In addition, flexible printed circuit board (FPCB) technology, currently the most commonly used for implementing flexible wireless biosensor systems, still cannot provide sufficient stretchability for electronic systems designed to be worn on the skin. Therefore, the attention of researchers is currently focused on creating the most reliable and flexible Bluetooth communication modules created using other technologies, and work in this area continues [265,266].

**Figure 13:**
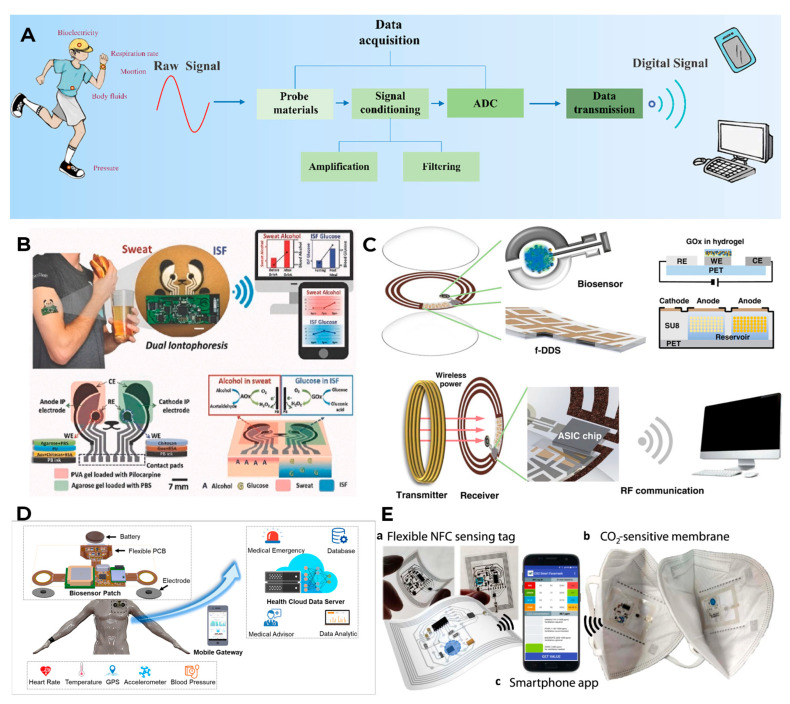
Data collection and transmission using wireless technologies in wearable biosensors. (**A**) Working processes of wearable sensors capable of tracking various signals of the human body, ranging from biophysical signals (including human movements, respiratory rate, bioelectricity, etc.) to biochemical signals (such as biological fluids, blood components, glucose, etc.). Reproduced with permission: copyright 2022, MDPI [256]. (**B**) Tattoo-based dual biofluid (sweat and interstitial fluid) sensing platform with hydrogel coverage as reservoir for electrochemical sensing as well as skin protector. Reproduced with permission: copyright 2018, Wiley [271]. (**C**) Schematic illustration of the smart contact lens for diabetic diagnosis and therapy. The smart contact lens is embedded with a biosensor, an f-DDS, a wireless power transmission system from a transmitter coil to a receiver coil, an ASIC chip, and a remote communication system as a ubiquitous platform for various diagnostic and therapeutic applications. Reproduced with permission: copyright 2020, AAAS [205]. (**D**) Wearable biosensor patch with IoMT application using Bluetooth low-energy module for transmission. Reproduced with permission: copyright 2022, MDPI [224]. (**E**) Smart facemask with integrated flexible NFC tag for wireless CO_2_ monitoring. Reproduced with permission: copyright 2022, Nature Research [272].

Radio frequency (RF) communication technology in wearable biosensors is most often used in the frequency range of 1 kHz-1000 MHz and provides data transmission over a distance of up to 20 m. In the study [267], it was shown that when sending mini-sensor data to a mobile phone with a transmission frequency of 1 Hz, the total power consumption of the microcontroller reached 15 MW. However, radio frequency communication technologies in various frequency ranges are highly dependent on the availability of special microchips or various commercial transmitters in both the transmitting and receiving devices. Therefore, it is difficult to use this technology to create a communication channel with users’ personal devices such as smartphones. NFC, a form of RFID, reduces both the power consumption of wearable devices and their size [268]. The NFC approach divides the wireless biosensor system into two parts: a flexible sensor that is worn on the body and an RFID reader that is located outside the body. For example, in 2020, a smart contact lens with an immunosensor for cortisol detection with a detection limit of 10 pg/mL was proposed, using an NFC antenna that operated at 13.56 MHz [269]. In 2023 [270], a glucose sensor based on the composite GOx/PEDOT:PSS/AuNPs/Prussian Blue was developed, controlled by a wireless NFC potentiostat. Some examples of how data are collected and transmitted using wireless technologies in wearable biosensors are shown in Figure 13.

In general, regardless of which specific technologies are used to connect the sensor to processing devices, Bluetooth, Wi-Fi, or RFID, all of them may be more or less susceptible to interference from other electronic devices, atmospheric conditions, and physical obstacles. In addition, possible security vulnerabilities that can lead to leaks of sensitive personal medical data of patients remain a serious problem for wearable biosensors using these communication technologies [273]. Therefore, all sensor data must be encrypted both during transmission and at rest. To ensure that data are unreadable even if they are intercepted during transmission, end-to-end encryption can be used using standards such as the Advanced Encryption Standard (AES) for data encryption and Diffie-Hellman Key Exchange (DH) for key pairing [274]. However, even in this case, doubts remain about how exactly the data obtained from wearable medical devices can be used, given their ability to track and control human movements and actions. Moving forward, the development of this aspect of wearable medical devices should include improved sensor security systems, increased user autonomy regarding their medical data, and legislative regulation of the collection and use of wearable device users’ medical data.

## 6. External Problems: Scaling, Certification, and Security

Finally, the last barrier that must be overcome for the successful implementation of a wearable device in healthcare is its transition from the laboratory level of a prototype or experimental sample to commercial mass production. This aspect includes both the technical issues of the transition from the laboratory to the plant, characterized by a sharp increase in the number of manufactured devices and automation of this process, as well as issues related to clinical trials, medical certification, and the direct launch of sales of a medical device in various markets. We should not forget about cybersecurity and privacy concerns: personalized medical devices are a source of increased risk [275], as they continuously collect users’ personal medical information in real time, which is considered more sensitive than other types of data [276]. Lastly, there is a need for some serious long-term work with potential users of new devices: the difficulties of people adopting new technologies and doubts about how well it really works can really mess with how well products are promoted and popularized.

In order to better assess all the possible problems that manufacturers of wearable devices may face at this stage, let us follow the entire path that an experimental biosensor sample needs to go through before it hits the counter. Again, we will divide this into two stages: the direct mass production of the device and its adaptation and certification for various markets.

First of all, all manufacturers pay attention to the cost of devices: it should be minimal in order to facilitate their commercialization. One of the simplest ways to reduce the cost of a batch of devices is to scale up their production. Production processes should be designed in such a way that they can be easily adjusted and scaled, thereby optimizing the efficiency of mass production. Fortunately, the market for wearable medical devices is quite large; its growth in the post-pandemic years has been driven by increasing attention to health management issues and the desire for a healthier lifestyle. In the future, it should cover the entire population of the Earth; therefore, from this point of view, the scalability of wearable biosensors is unlimited. However, one of the main obstacles in the development and mass production of wearable medical sensors (as well as any other flexible electronics) is ensuring the stability and uniformity of material characteristics and operating parameters of devices at high production volumes [277]. Each sensor must have the same sensitivity and lifetime. For this, separate automated testing lines and mass calibration procedures must be provided; otherwise, large deviations between batches of devices are inevitable, which is unacceptable for medical equipment. This increases the cost per unit, which includes other factors such as the high cost of flexible conductive materials and nanomaterials, which are necessary for the efficient operation of miniature sensors [278]. In addition, complex manufacturing methods for such devices often require special equipment and a strictly controlled environment, specific cleaning, or storage conditions. For example, polymer-based microfluidic layers often used in wearable biosensors should be manufactured using technologies used in clean rooms and with the predominant use of non-biodegradable polymer substrates [279]. The conductive layer of biosensors is also often applied to the surface of polymer substrates using a combination of expensive components (metal nanoparticles and carbon ink) and complex deposition methods such as lithography, electron beam evaporation, spraying, or inkjet printing [280]. Finally, the use of a biological layer in wearable biosensors requires the involvement of specialists in the field of biotechnology, as well as the introduction of expensive stages of preparation and purification of the biological component (purification of enzymes, covalent immobilization of antibodies, etc.). At the same time, wearable biosensors must have a self-calibration function to avoid time-consuming procedures such as pre-sampling or user and medical staff training. All these costs in the production of wearable medical devices ultimately affect the pricing policy of the finished device, which, in turn, may lead to the inaccessibility of devices for some segments of the population or even some geographical regions.

The multistage and multifaceted nature of the biosensor creation process directly affects the complexity of building a technological map for biosensor production: often, a finished device cannot be manufactured within the framework of not only one department but also within a single production complex. This is another difficulty in the transition of biosensors from the laboratory level to mass production: the processing of “manual” protocols for mass equipment, the need for strict coordination of logistics, and the selection of reliable subcontractors with relevant experience in medical electronics/biomaterials. The development of biosensors requires an uninterrupted supply of many specialized components, such as specific enzymes and antibodies, hypoallergenic biocompatible materials, or integrated circuits. In such circumstances, one of the few ways to reduce the cost of developing and releasing devices is to optimize and standardize production processes as much as possible. The trend towards simplifying technologies and adapting biosensors to mass production is also observed in the academic literature, even among researchers who are not directly involved in the production of ready-made medical devices. Recent reviews of wearable biosensors show that the creators of modern electrochemical and optical sensors using microfluidic structures are paying increased attention to the compatibility of their developments, with low-cost and high-performance production processes [281,282]. Nevertheless, the roadmap for the transition from the laboratory to the plant is absolutely unique for each specific device, and at the moment, there is still no “universal” approach.

However, one should not think that scaling up production and building reliable supply chains is the most difficult stage in the production of medical devices. Manufacturers often have much more problems with the stages of clinical trials, registration, and certification of their devices in different regions, as well as developing effective distribution and pricing strategies for their devices. Even a perfectly manufactured medical device is useless without proof of its effectiveness and safety. Clinical trials and subsequent registration for new medical devices are the most formalized and often the longest stage [283,284]. In most jurisdictions, clinical trials must be completed for each device that will be further marketed as a medical device. At the same time, the structure and requirements for clinical trials of wearable medical devices differ significantly from the standards adopted in traditional biopharmaceutical research and development, which is why there is currently no single “gold standard” for methodological approaches to conducting clinical trials for wearable biosensors [285]. In 90% of Phase III clinical trials of pharmaceutical drugs, double-blind randomized trials can be seen in which the results are evaluated with the participation of thousands of subjects who have been monitored for many months or years. However, in the case of wearable biosensors, much more flexibility in test design and statistical analysis is often needed. Thus, the Dexcom G5 Mobile CGM device became the first CGM device approved by the FDA in 2016. The developers needed to conduct two clinical trials involving 130 adults and children aged 2 years and older with diabetes. During the research, the readings of the system were compared with the results of measurements of a glucose meter and laboratory tests for seven days [286]. Ideally, clinical trials should include as diverse a group of participants as possible: by age, gender, ethnicity, and with various comorbidities. A sensor calibrated on healthy 25-year-old men may malfunction in an elderly woman with diabetes or skin problems. This significantly complicates and increases the cost of recruiting patients. Moreover, conducting a large-scale double-blind trial of an implantable or semi-implantable device may simply be impossible, since using a fictitious control for a target group of patients may be unethical or simply dangerous due to the risk associated with the implantation or procedure itself [221]. As a result, device trials are much less likely to be blinded or randomized than drug trials. For some devices, there are possibilities to use alternative test methods, such as comparison with existing analogues or the use of mathematical modeling methods, which allows manufacturers and regulatory authorities themselves to get a good understanding of the risks and benefits of the device without the need for detailed tests [287]. Currently, dozens of devices for long-term monitoring of metabolites are offered on the market, the most famous of which are shown in Table 1.

The data presented in the table clearly show that most sensor devices on the market are related to glucose measurement. This can be explained primarily by the continuous growth in the number of diabetes patients worldwide and the market volume for these devices. Since demand creates supply, it is primarily profitable for large manufacturers to direct funding to research in the field of glucose sensors, as they are sure to find their end users. In addition, in many cases, such sensors are a necessity, which encourages consumers to purchase these devices. Wearable sensors for measuring lactate, alcohol, or ketones are used in rarer cases and do not significantly improve the quality of life for the end user. Therefore, their sales volume is not as high, which, in turn, does not sufficiently encourage large investors and manufacturers to spend on research and development, certification, and medical research.

Each country or region also has its own unique requirements, documentation, and certification procedures. Devices that are classified as “medical devices” by regulatory authorities in the United States, the United Kingdom, and the EU must generally be manufactured, certified, tested, monitored, and distributed in accordance with a quality management system approved by the relevant government regulatory authorities. This includes any software, embedded hardware, as well as data collection and transmission carried out by the device [294]. In the United States, all medical devices are classified into three classes, depending on the level of risk to the patient. Most wearable biosensors designed for medical purposes fall into Class II, which requires a 510(k) procedure. As part of this procedure, the manufacturer must prove that it has similar technical characteristics compared to the previous (legally sold) device, which uses a similar engineering solution [295]. For fundamentally new devices that have no analogues, there is a more complex and expensive De Novo or Premarket Approval (PMA) path for high-risk devices (Class III). It is important to note that devices designed for general wellness and not claiming to diagnose or treat specific diseases can be classified as “low-risk wellness devices” and are not subject to the strict FDA requirements for medical devices. At the same time, it should be noted that the pace of development of information technology and technology in general forces the FDA to constantly update its regulations on medical devices and regularly adapt to changes [284]. So, in 2017, the FDA effectively allowed the use of real-world evidence to justify decisions on the regulation of medical devices [296]. Since the publication of these guidelines, many manufacturers of new medical devices have considered collecting real-world data as the main way to register their devices instead of traditional data sourced from randomized clinical trials [297].

The rules for the EU are different from those of the U.S. For sale in the European Union, medical devices must have a label confirming their compliance with the requirements of the Medical Device Regulation (MDR), which entered into force in May 2017 and became applicable on 26 May 2021 [298]. The process also includes the classification of devices according to the degree of risk and, for devices above Class I, requires the participation of an authorized institute (for conformity assessment) [299]. In addition to complying with regulatory requirements for medical devices, manufacturers of wearable devices in the EU must also comply with other requirements: the General Data Protection Regulation (GDPR) [300], regulatory requirements in the field of cybersecurity [301], and the Artificial Intelligence Act, which regulates the use of artificial intelligence [302]. The issue of personal data protection should also be approached with special care, and it is not just about the legislative acts regulating it. With the increasing integration of wearable biosensors into health monitoring systems, data security and privacy have come under intense scrutiny [303]. The information collected through the integration of wearable devices ranges from demographic data such as age, gender, and location to more detailed data related to people’s health and well-being [304]. People will trust wearable medical devices only if reliable data transmission and confidentiality are ensured. Medical data are some of the most vulnerable, so any data leak can not only lead to huge fines but also completely destroy the company’s reputation and the trust of patients [204]. Therefore, any WMD in the modern world needs additional reliable security measures to prevent violations or unauthorized access to the data of such users [278].

Medical devices in India have their own classification [305]: devices are divided into four risk classes, while the software must be certified in the same class as the associated device. It is worth noting that these are just three markets out of more than a hundred potentially open to wearable medical devices. In some countries, like India, there are simplified requirements for obtaining a license to import medical devices from other countries, but in most countries, additional local clinical trials will be required for high-risk devices [306]. Manufacturers planning to expand the global reach of their devices need to carefully consider the requirements of each individual country in which they plan to sell them. Moreover, new and stricter requirements for medical devices are being introduced every year, which inevitably leads to an increase in the complexity and cost of certification. Finally, one should not underestimate the impact of political processes and lobbying by large established manufacturers on the potential certification of certain devices in the markets. The government of a particular country can either speed up the approval of appropriate devices (in the case of national diabetes control programs, for example) or simply block the product from entering the market as part of trade wars or sanctions.

In addition to the political and bureaucratic difficulties that manufacturers of wearable biosensors may face in each individual country, they also need to consider the pricing and availability of devices in each individual region. And here, social aspects are added to the purely economic aspect. Theoretically, wearable biosensors represent very promising tools for achieving health equity, which is defined as “a condition in which everyone has a fair and equal opportunity to achieve the highest level of health” [307]. The authors who study this issue note that barriers to achieving equality in healthcare include poverty, institutionalized racism, environmental factors, and geography [308]. Therefore, in an ideal world, biosensors should not only be accurate, reliable, and safe but also accessible to different population segments. All this complicates the issue of end device pricing: manufacturers need to find a balance between recouping the huge costs of development and testing and accessibility for the end user. The high price will make the device an elite product, accessible only to the well-off segments of the population. This can be seen in the example of existing systems for CGM, the constant use of which places a heavy financial burden on patients [309]. In developed countries, this can be circumvented by using reimbursement models, where part of the cost of a wearable biosensor for the end user will be covered by public or private insurance. However, there are two problems here, too. The first is that to use such a scheme, manufacturers need to prove to insurance companies and ministries of health that the cost of a biosensor will save the system more money in the long run (for example, by preventing costly complications). This is a separate area of complex research that requires additional investments, which is at odds with the company’s original goal [310]. Secondly, such a scheme is impossible in many developing countries, where states, like the population, simply do not have the opportunity to finance such programs. Therefore, the entire burden of adapting devices to new markets falls on the manufacturers themselves, including not only the localization of the interface, instructions, and customer support systems but also often the development of educational programs for patients and training courses for clinical staff [311]. When adopting new devices, it is essential to consider the cultural aspects of each individual country, which are not always obvious at first glance. For example, the authors of some reviews point to the existence of a certain social stigma on the wearing of medical devices on prominent body parts in the Asia-Pacific region [312]. Overcoming such barriers is possible only with the help of effective and well-coordinated joint educational work of manufacturers and government agencies. Do not forget about other social factors: wearable biosensors almost certainly require the end user to have a smartphone, stable Internet, and the ability to use applications in order to work effectively. This automatically excludes a significant portion of the elderly and low-income people or those living in remote areas. As a result, some large manufacturers (for example, in the CGM market) deliberately ignore this layer of potential users, not even entering the markets of many developing countries in Asia and Africa [313] and not adapting devices to facilitate their use, for example, by older users, since the costs of this will not be recouped by the potential benefits from these markets. However, as diabetes prevalence rates continue to rise, research efforts and interventions to reduce price barriers are expected to contribute to the development of the CGM market, and with it, the entire market for wearable medical devices [314].

## 7. Conclusions

**Figure 14:**
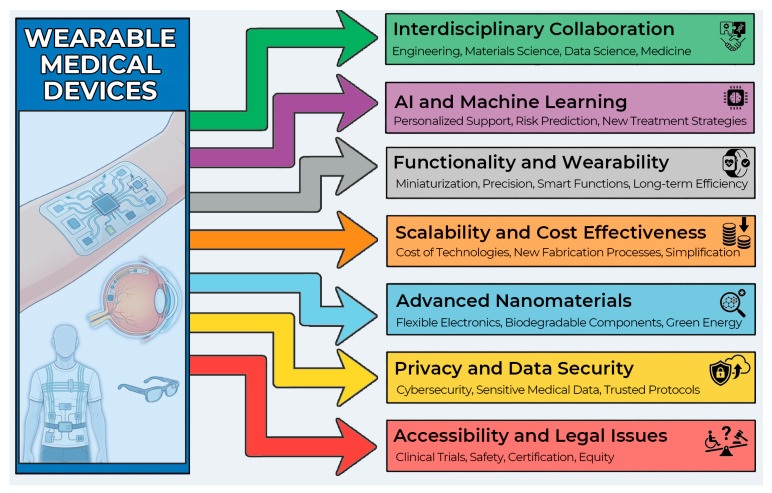
The main directions that developers of wearable medical devices need to pay attention to in the near future.

Wearable medical devices have already significantly improved the quality of life of many patients by facilitating non-invasive real-time monitoring of various diseases. Despite the enormous possibilities of using wearable biosensors in medicine, there are a number of technical, social, and economic challenges that need to be solved in order to make such devices more efficient, reliable, and widespread. The main directions in which the field of wearable medical devices will develop in the near future are shown in Figure 14. First of all, there are still many “laboratory” problems related to the detection range, efficiency, stability, and accuracy of the biosensors themselves. Equally important are the issues of reuse and recovery of wearable devices used for real-time monitoring. The active introduction of artificial intelligence and machine learning can radically change the functionality of biosensors, providing the ability to interpret complex biomarker profiles in real time, which should not only increase the accuracy of measurement but also expand the scope of their application. It will also be very important to solve the problems of scalability and cost effectiveness. Developing reliable, highly sensitive, and energy-efficient devices remains an extremely expensive task, which limits the possibilities for advancing wearable medical devices. From an electronics perspective, the development of flexible, durable, breathable, and biocompatible materials remains an important challenge to meet the stringent demands of wearable devices. Important areas for sustainable and low-cost mass production of wearable sensors remain the creation of disposable and replaceable components, efficient and autonomous ways to collect energy, including “green” power sources (such as disposable solar panels or biofuel cells), or powerless options using near-field contactless communication. This is necessary to solve the problem of recycling wearable sensors after their use, as currently, most of the components of these devices are still not biodegradable and pollute the environment. Finally, critical security and privacy issues remain, as wearable medical devices must provide the user with the opportunity to receive not only timely but also safe care. It is hoped that interdisciplinary collaboration between materials scientists, engineers, healthcare professionals, and government agencies will be crucial to the development of biosensors that are not only accurate, reliable, and scalable but also accessible to diverse populations around the world.

However, the long and difficult path to creating modern CGM systems, which are now used by millions of people around the world, shows that all these efforts will not be in vain in the end. The active introduction of wearable biosensors in the next decade may simply revolutionize healthcare, and with it, raise the quality of human life to a new level.

## Figures and Tables

**Table 1:** Examples of commercially available wearable biosensors for long-term health monitoring and some of their characteristics.

Name	Manufacturing Company	Photo	Key Features	Interfering Substances	Ref.
FreeStyle Libre^®^ 2	Abbott diabetes care, Alameda, CA, USA	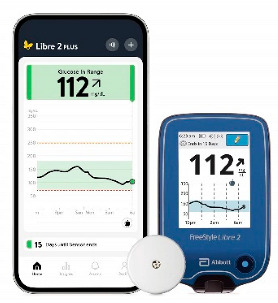	Flash glucose monitoring Can be used by children 4 years and older Sensor warm-up time is 12 h Alarm notifications within 6 m of the reading device	Ascorbic acid (>500 mg/day); salicylic acid (0.50 mmol/L)	[114]
FreeStyle Libre^®^ 3		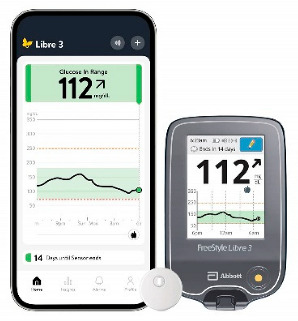	Continuous glucose monitoring Thickness of 0.35 mm 15 days of continuous operation IPX7 water protection	Ascorbic acid (>1000 mg/day)	
FreeStyle Navigator^®^ II		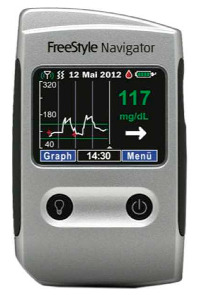	Finger measurement 30 m transmission range 94.2% clinically accurate readings Cannot be used in proximity to HAM radio equipment operating at 433.6 Mhz	Ascorbic acid; salicylic acid; ibuprofen	[288]
AiDEX^TM^	MicroTech Medical (Hangzhou) Co., Ltd., Hangzhou, China	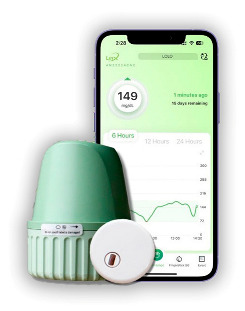	Sensor warm-up time is 1 h Weight 2.2 g Real-time Bluetooth data transmission IP68 durability and water resistance Flexible placement (upper arm/abdomen)	Triglycerides (>3000 mg/dL); cholesterol (>500 mg/dL)	[289]
Dexcom G7	Dexcom, San Diego, CA, USA	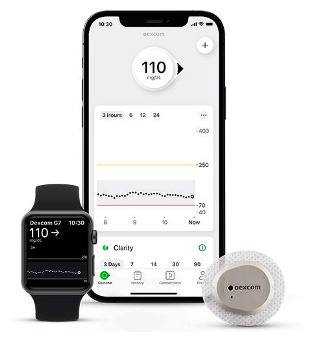	Sensor warm-up time is 30 min Real-time glucose readings every 5 min Remote monitoring for up to 10 followers Mean absolute relative difference of 8.2%	Hydroxyurea; acetaminophen (>4 g per day)	[290]
Eversense^®^ 365	Ascensia Diabetes Care, Basel, Switzerland	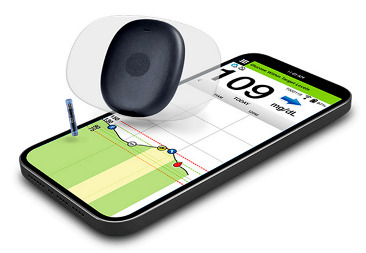	Sensor remains under the skin for 365 days Requires weekly calibrations Removable smart transmitter On-body vibration alerts	Tetracyclines; sorbitol; mannitol	[291]
Liipoo AbsolutSweat Hydration Biosensor	Shenzhen Refresh Biosensor Technology Co., Ltd., Shenzhen, China	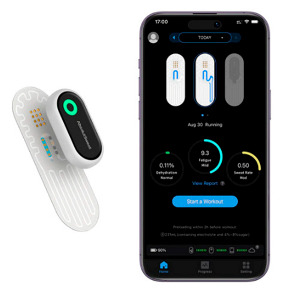	Provides on-body vibration alerts Information on water loss and electrolyte loss, glucose level, sweating, rate etc. Wearable on the chest		[292]
Nix	Boston, MA, USA, Nix	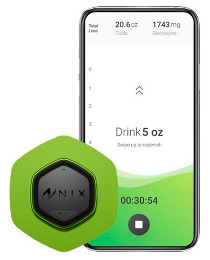	Real-time sweat analysis Individual hydration plans based on sweat composition and electrolyte loss rate Remote monitoring for coaches and trainers		[293]

## Data Availability

Data is contained within the article.
